# Molecular Pathology

**DOI:** 10.1186/s12967-024-04868-7

**Published:** 2024-01-22

**Authors:** Sarah Warren

**Affiliations:** Gilead Sciences, Seattle, WA USA

Accumulating scientific insights, along with novel technological advances, are leading to greater understanding of the underlying causes of disease and novel diagnostic assays to inform clinical care. To support continued development in the field, we are pleased to announce a new section in the *Journal of Translational Medicine* focused on Molecular Pathology.

This new section will serve as a platform to highlight studies that characterize human-derived biological materials, including both tissue and liquid biopsies, using molecular profiling techniques to elucidate the underlying causes of disease and impacts of therapeutic interventions. We expect this section to appeal to scientists working on transcriptomics, proteomics, lipidomics, glycomics and beyond. Furthermore, we anticipate most studies in this section will be preclinical or translational in nature, although we will consider works from all stages of fundamental and applied research. We also welcome manuscripts that describe new technology platforms that advance molecular profiling or novel computational approaches to support data analysis.

Molecular pathology has existed as a discipline for centuries, though without the modern name. The first reports of molecular characterization of a liquid biopsy are from 1764 when Domenico Cortugno connected the presence of protein in urine to dysfunction of the kidneys [1]. The field grew slowly for the next 200 years as scientific advances were focused on microscopy, but the development of novel methods for molecular profiling were quickly applied to the study of disease. Notably, immunofluorescence techniques invented by Albert Coons in 1961permitted detection of proteins in cells and tissues, [2] and in 1975, Edwin Southern invented the Southern blot to hybridize probes to specific DNA sequences and used the technique to identify a particular genetic polymorphism co-occurring with X-linked retinitis pigmentosa [3]. Two decades later, molecular pathology was revolutionized by the advent of high throughput technologies such as microarrays and RNA sequencing in the 1990s that permitted deep classification and characterization of many samples in parallel [4], and the field has continued to progress rapidly.

These advances in technologies have led to deeper understanding of the underlying causes of disease. Genomic profiling of tumor tissue across a variety of indications has revealed mutations in specific tumor driver pathways, such as BCR-ABL, KRAS, ERBB2, MET, BRAF, NTRK, which have led to the development of therapeutics directed against these targets [5]. The field of immuno-oncology has benefitted immensely by transcriptional profiling of tumor that revealed an intrinsic immunological status that correlates with better prognosis and response to immunological agents [6], and derivations of this signature have also been developed into diagnostic assays. The characterization of tumor burden, whether by circulating tumor DNA, circulating tumor cells, or molecular signatures of disease, have also been deployed clinically to inform patients about disease progression and prognosis, especially in the setting of hematological malignancies [7]. Recent advances in molecular pathology, especially the development of profiling systems with higher resolution (including spatial resolution), higher plex, and ability to characterize multiple analytes from a single sample, are continuing to push the field forward. Parallel advances in statistical methods and artificial intelligence/machine learning approaches for data analysis will also drive greater biological insights and ultimately advances in patient care. As the field continues to progress, we expect to see further advances across multiple areas, especially in profiling liquid biopsies that simplify sample collection and facilitate longitudinal testing to monitor disease progression. We also expect the insights from molecular profiling to translate into novel diagnostic assays that further identify patient populations likely to respond to a given therapeutic.

To support innovation in the field of molecular profiling, this new section of *Journal of Translational Medicine* is committed to raising visibility for impactful studies in the field. We are aligned will the goals of the journal to provide an open access platform with an expedient review process. The section editorial board consists of highly qualified experts in the field who will provide thoughtful consideration of submissions, and our diverse pool of reviewers have a wide variety of backgrounds and perspectives that will contribute to useful feedback to authors. In conclusion, we welcome manuscript submissions to this new section on molecular pathology and we look forward to promoting advances in the field.

## References

[1] Schena FP (1994). Domenico Cotugno and his interest in proteinuria. Am J Nephrol..

[2] Coons A (1961). The beginnings of immunofluorescence. J Immunol.

[3] Bhattacharya SS (1984). Close genetic linkage between X-linked retinitis pigmentosa and a restriction fragment length polymorphism identified by recombinant DNA probe L1.28. Nature.

[4] Lowe R (2017). Transcriptomics technologies. PLoS Comput Biol.

[5] Waarts MR (2022). Targeting mutations in cancer. J Clin Invest.

[6] Wang E, Worschech A, Marincola FM (2008). The immunologic constant of rejection. Trends Immunol.

[7] Dekker SE (2023). Using measurable residual disease to optimize management of AML, ALL, and chronic myeloid leukemia. Am Soc Clin Oncol Educ Book.

